# Predicting hypertension by obesity- and lipid-related indices in mid-aged and elderly Chinese: a nationwide cohort study from the China Health and Retirement Longitudinal Study

**DOI:** 10.1186/s12872-023-03232-9

**Published:** 2023-04-20

**Authors:** Yuqing Li, Jiaofeng Gui, Xiaoyun Zhang, Ying Wang, Yujin Mei, Xue Yang, Haiyang Liu, Lei-lei Guo, Jinlong Li, Yunxiao Lei, Xiaoping Li, Lu Sun, Liu Yang, Ting Yuan, Congzhi Wang, Dongmei Zhang, Huanhuan Wei, Jing Li, Mingming Liu, Ying Hua, Lin Zhang

**Affiliations:** 1 Department of Graduate School, Wannan Medical College, 22 Wenchang West Road, Higher Education Park, Wuhu City, An Hui Province People’s Republic of China; 2 Student Health Center, Wannan Medical College, 22 Wenchang West Road, Higher Education Park, Wuhu City, An Hui Province People’s Republic of China; 3 Department of Surgical Nursing, School of Nursing, Jinzhou Medical University, No.40, Section 3, Songpo Road, Linghe District, Jinzhou City, Liaoning Province People’s Republic of China; 4 Department of Occupational and Environmental Health, Key Laboratory of Occupational Health and Safety for Coal Industry in Hebei Province, School of Public Health, North China University of Science and Technology, Tangshan, Hebei Province People’s Republic of China; 5 Obstetrics and Gynecology Nursing, School of Nursing, Wannan Medical College, 22 Wenchang West Road, Higher Education Park, Wuhu City, An Hui Province People’s Republic of China; 6 Department of Emergency and Critical Care Nursing, School of Nursing, Wannan Medical College, 22 Wenchang West Road, Higher Education Park, Wuhu City, An Hui Province People’s Republic of China; 7 Department of Internal Medicine Nursing, School of Nursing, Wannan Medical College, 22 Wenchang West Road, Higher Education Park, Wuhu City, An Hui Province People’s Republic of China; 8 Department of Pediatric Nursing, School of Nursing, Wannan Medical College, 22 Wenchang West Road, Higher Education Park, Wuhu City, An Hui Province People’s Republic of China; 9 Department of Surgical Nursing, School of Nursing, Wannan Medical College, 22 Wenchang West Road, Higher Education Park, Wuhu City, An Hui Province People’s Republic of China; 10 Rehabilitation Nursing, School of Nursing, Wanna Medical College, 22 Wenchang West Road, Higher Education Park, Wuhu City, An Hui Province People’s Republic of China

**Keywords:** Hypertension, Cohort study, Middle-aged and elderly, Receiver operating characteristic curve

## Abstract

**Background:**

Currently, the study outcomes of anthropometric markers to predict the risk of hypertension are still inconsistent due to the effect of racial disparities. This study aims to investigate the most effective predictors for screening and prediction of hypertension (HTN) in the Chinese middle-aged and more elderly adult population and to predict hypertension using obesity and lipid-related markers in Chinese middle-aged and older people.

**Methods:**

The data for the cohort study came from the China Health and Retirement Longitudinal Study (CHARLS), including 4423 middle-aged and elderly people aged 45 years or above. We examined 13 obesity- and lipid-related indices, including waist circumference (WC), body mass index (BMI), waist-height ratio (WHtR), visceral adiposity index (VAI), a body shape index (ABSI), body roundness index (BRI), lipid accumulation product index (LAP), conicity index (CI), Chinese visceral adiposity index (CVAI), triglyceride-glucose index (TyG-index) and their combined indices (TyG-BMI, TyG-WC, TyG-WHtR). To compare the capacity of each measure to forecast the probability of developing HTN, the receiver operating characteristic curve (ROC) was used to determine the usefulness of anthropometric indices for screening for HTN in the elderly and determining their cut-off value, sensitivity, specificity, and area under the curve (AUC). Association analysis of 13 obesity-related anthropometric indicators with HTN was performed using binary logistic regression analysis.

**Results:**

During the four years, the incident rates of HTN in middle-aged and elderly men and women in China were 22.08% and 17.82%, respectively. All the above 13 indicators show a modest predictive power (AUC > 0.5), which is significant for predicting HTN in adults (middle-aged and elderly people) in China (*P* < 0.05). In addition, when WHtR = 0.501 (with an AUC of 0.593, and sensitivity and specificity of 63.60% and 52.60% respectively) or TYg-WHtR = 4.335 (with an AUC of 0.601, and sensitivity and specificity of 58.20% and 59.30% respectively), the effect of predicting the incidence risk of men is the best. And when WHtR = 0.548 (with an AUC of 0.609, and sensitivity and specificity of 59.50% and 56.50% respectively) or TYg-WHtR = 4.781(with an AUC of 0.617, and sensitivity and specificity of 58.10% and 60.80% respectively), the effect of predicting the incidence risk of women is the best.

**Conclusions:**

The 13 obesity- and lipid-related indices in this study have modest significance for predicting HTN in Chinese middle-aged and elderly patients. WHtR and Tyg-WHtR are the most cost-effective indicators with moderate predictive value of the development of HTN.

## Background

Hypertension (HTN) is a chronic noncommunicable condition in which the Systolic blood pressure (SBP) and/or Diastolic blood pressure (DBP) are elevated, frequently accompanied by functional or physical damage to the heart, brain, kidney, and other organs [1]. One of the most common chronic non-communicable diseases in the world, HTN is also a major risk factor for cardiovascular disease, with HTN complications accounting for approximately 53% of all cardiovascular disease-related deaths [2]. According to current statistics, the global incidence of HTN will climb from 26% in 2000 to 29.2% by 2025 [3]. Although HTN is more prevalent in higher-income countries such as the United States [4], it is quickly growing in low- and middle-income countries [5, 6]. According to hypertension survey data from 2012 to 2015, the incident rate of hypertension among adults in China was 23.2% [7], and the number of fatalities caused by hypertension among Chinese residents was 2.54 million in 2017, with cardiovascular disease deaths accounting for 95.7% [8].

Obesity has become an increasingly important worldwide public health concern as people's living standards have improved and their life rhythms have accelerated. Weight increase and blood pressure rise are clearly linked, according to studies [9–11], and these obesity markers have a strong dose–response association with the onset of hypertension [12]. In previous studies, body mass index (BMI) was used as the most prevalent marker of obesity and overweight [13]. In recent years, scholars have proposed many new obesity and lipid-related indicators, but the results of studies on anthropometric markers to predict hypertension risk remain inconsistent due to ethnic differences and various factors [14–24]**.** However, these studies are not representative for predicting hypertension in Chinese middle-aged and older adults because most of these studies describe only one indicator and do not compare it with other indicators.

Therefore, this study's objective was to investigate the screening and predicting functions of obesity and lipid-related indicators for HTN in middle-aged and elderly Chinese, as well as the ideal predicted cut-off value to provide a basis for HTN prevention and therapy.

## Methods

### Study design and setting

The data for our analysis came from the 2011 China Health and Retirement Longitudinal Survey Wave (CHARLS Wave 2011), a nationally representative longitudinal investigation, which was conducted by the China Center for Economic Research at Peking University [25]. Individuals without HTN in baseline from the CHARLS Wave 2011 study were included in our analysis after missing data subjects were removed. Subsequently, data collection was conducted in 2015. The numbers of individuals who completed both the baseline and follow-up surveys were 4423 for the cohort design. Without any direct interaction with people, all data are provided in the open as microdata at http://charls.pku.edu.cn/index/zh-cn.html. All participants gave their informed consent prior to the collection of data, and the study was approved by the Ethics Committee of the China Center for Economic Research at Peking University.

### Individuals

Study subjects for this investigation were chosen from the China Health and Retirement Longitudinal Study (CHARLS), Wave 1 (2011). The CHARLS Wave 2011 was used to choose participants for this study [25]. This research is a cohort study. Patients with undetected hypertension were included to our follow-up group in 2011. In 2015, the incident rate of individuals impacted by 13 indicators was evaluated four years later. The average age of the 4,423 individuals participating in CHARLS was 57.43 years (standard deviation SD = 8.89, range 45–98 years). Males had a mean age of 59.08 years (SD = 8.79, range 45–98 years) while females had a mean age of 56.02 years (SD = 8.74, range 45–90 years).

### Baseline characteristics

Baseline characteristics including age, sex (1 = male; 2 = female), education (1 = illiterate; 2 = less than elementary school; 3 = high school; 4 = above vocational school), marital status (1 = married; 2 = single), living place (1 = rural; 2 = urban), smoking status (1 = no; 2 = former smoke; 3 = current smoke), drinking status (1 = no; 2 = less than once a month; 3 = more than once a month), taking activities (1 = no; 2 = yes), and Having regular exercises (1 = no; 2 = less than exercises; 3 = regular exercises),and the counts of Chronic diseases (0 = 0; 1 = 1–2; 2 = 3–14)were collected by self-report. Most variables depended on our previous research studies [26–31].

### Definition

Participants were divided into hypertensive and non-hypertensive groups. Hypertension was formerly described as having a systolic blood pressure (SBP) ≥ 140 mmHg and/or a diastolic blood pressure (DBP) ≥ 90 mmHg, or with hypertension diagnosed by self-reported physician diagnosis. Non-hypertension was defined as SB*P* < 140 mm Hg, DB*P* < 90 mmHg and patients who have not received antihypertensive treatment. This classification was widely used in our previous studies [26, 28, 31–33].

### Glucose, High-density lipoprotein cholesterol, Triglycerides Measurement

The Chinese Center for Disease Control and Prevention in Beijing received the venous blood samples within two weeks of them leaving the Centers for Disease Control and Prevention station. The samples were immediately stored and frozen at -20 °C before being delivered. When the necessary assays were completed in the lab of the Chinese Medical University, they were put in a deep refrigerator and kept at -80 °C. At the Capital Medical University Youanmen Clinical Laboratory, triglycerides (TG), fasting plasma glucose (FPG), and high-density lipoprotein cholesterol (HDL-C) were measured using the enzyme colorimetric assay. We divided TG levels into two groups, < 150 mg/dL and ≥ 150 mg/dL, in accordance with a classification that has previously been employed in studies [26]. When fasting plasma glucose is ≥ 126 mg/dl or 7.0 mmol/dl and above, it indicates abnormal blood glucose [33]. HDL-C values lower than 40 for men and 50 mg/dL for women were considered abnormal [34].

### Measurements

Omron™ HEM-7112 Monitor (Manufacturer: Omron Co., Ltd., Dalian, China) was used to monitor blood pressure on the respondent's left arm three times at 45-s intervals. Respondents were asked to sit with both feet on the floor and their left arm comfortably supported, palm up. Unless they were wearing a short sleeve or a flimsy shirt, respondents were requested to roll their sleeves up. The cuff's bottom was about half an inch above the respondent's elbow, and the air tube went down the center of the respondent's arm. After the interviewer presses the start button, the cuff automatically inflates, and then deflates to show systolic and diastolic blood pressure. After recording the results truthfully, the interviewer uses a stopwatch and waits for 45–60 s before starting the next measurement.

BMI was computed by dividing body weight (kg) by height (m) squared [35]. At the conclusion of expiration, the umbilical level was chosen, and the waist size was measured [36]; when the waist circumference(WC) of women ≥ 85 cm, and men ≥ 90 cm, known as central obesity. Waist-height ratio (WHtR) was calculated by the ratio of WC to height [37]. Visceral adiposity index (VAI) was calculated using BMI, WC, TG and HDL-C, with different formulas for men and women [38]. It is important to note that VAI, Chinese visceral adiposity index (CVAI), lipid accumulation product index (LAP), and triglyceride-glucose (TyG) index were required to perform invasive manipulations to obtain TG and HDL-C. Other indicators were calculated using the following equations [18, 39–45].

1 $$\mathrm{BMI}=\frac{Weight}{{Height}^{2}}$$

2 $$\mathrm{WHTR}=\frac{WC}{Height}$$

3 $$\begin{array}{c}\mathrm{Males}:\mathrm{VAI}=\frac{WC}{39.68+(1.88\times BMI)}\times \frac{TG}{1.03}\times \frac{1.31}{HDL}\\ \mathrm{Females}:\mathrm{VAI}=\frac{WC}{39.58+(1.89\times BMI)}\times \frac{TG}{0.81}\times \frac{1.52}{HDL}\end{array}$$

4 $$\mathrm{ABSI}=\frac{WC}{{Height}^\frac{1}{2}\times {BMI}^\frac{2}{3}}$$

5 $$\mathrm{BRI}=\sqrt[364.2-365.5]{1- \left(\frac{WC\div {(2\pi )}^{2}}{{(0.5\times Height)}^{2}}\right)}$$

6 $$\begin{array}{c}\mathrm{Males}:\mathrm{LA}P=\left[\mathrm{WC }\left(\mathrm{cm}\right)-65\right]\times \mathrm{TG }(\mathrm{mmol}/\mathrm{l})\\ \mathrm{Females}:\mathrm{LA}P=\left[\mathrm{WC }\left(\mathrm{cm}\right)-58\right]\times \mathrm{TG }(\mathrm{mmol}/\mathrm{l})\end{array}$$

7 $$\mathrm{CI}=\frac{WC(m)}{\sqrt[0.019]{\frac{weight(kg)}{height(m)}}}$$

8 $$\begin{array}{c}\mathrm{Males}:CVAI=-267.93+0.68\times age+0.03\times BMI(kg/m2)+4.00\times WC(cm)+22.00\times Log10TG(mmol/l)-16.32\times HDL-C(mmol/l)\\\mathrm{Females}:CVAI=-187.32+1.71\times age+4.32\times BMI(kg/m2)+1.12\times WC(cm)+39.76\times Log10TG(mmol/l)-11.66\times HDL-C(mmol/l)\end{array}$$

9 $$\mathrm{TyG}\;\mathrm{index}=\mathrm{Ln}\lbrack(\mathrm{TG}(\mathrm{mg}/\mathrm{dl})\times\mathrm{glucose}(\mathrm{mg}/\mathrm{dl})/2)\rbrack$$

10 $$\mathrm{TyG}-\mathrm{BMI}=\mathrm{ TyG}\times \mathrm{BMI}$$

11 $$\mathrm{TyG}-\mathrm{WC}=\mathrm{ TyG}\times \mathrm{WC}$$

12 $$\mathrm{TyG }-\mathrm{WHtR}=\mathrm{ TyG}\times \mathrm{WHtR}$$

### Statistical analysis

Statistical Product Service Solutions (SPSS) software, version 25.0, was used to conduct the analyses (IBM SPSS, Armonk, NY, USA). By sex, sociodemographic traits were analyzed and percentages were provided. To compare the categorical variable distribution across sex, a chi-square test was utilized. The mean and standard deviation were used to express continuous variables. In order to evaluate the variations in mean distributions by sex, independent samples t-tests were utilized. The unadjusted and adjusted relationships between anthropometric and HTN were evaluated using binary logistic regression. We calculated odds ratios (ORs) and 95% confidence intervals (95%CI) adjusting for age, educational levels, marital status, live place, current smoking, alcohol drinking, activities, exercises, and chronic diseases. To determine the area under the curve (AUC) and 95% confidence interval as a predictor of hypertension, the receiver operating characteristic curve (ROC) was utilized [46]. The significance of the area under the curve is that an area greater than 0.9 indicates high accuracy, 0.7–0.9 indicates moderate accuracy, 0.5–0.7 indicates low accuracy, and 0.5 indicates a chance result [47]. The ROC curve can also be used to determine sensitivity, specificity, positive predictive value, negative predictive value, positive likelihood ratio, and negative likelihood ratio. The Youden index, which is derived using the formula: [maximum (sensitivity + specificity-1)] [48], which is the maximum vertical distance between the ROC curve and the diagonal or chance line, determines the cut-off value of the predictor based on the highest value. Data were analyzed using chi-square test followed by Bonferroni correction to reassess the significance level.

## Results

Table 1 shows the basic characteristics of the participants. A total of 4,423 subjects were included in this study, of whom 2038(46.10%) were male and 2385(53.90%) were female. Among them, there were significant differences between men and women in age, education, marital status, alcohol consumption, smoking, BMI, WHtR, VAI, ABSI, body roundness index (BRI), LAP, conicity index (CI), CVAI, TyG index, TyG-BMI, TyG-WC, TyG -WHtR (*P* < 0.05). However, the current residence, number of chronic diseases, exercise, taking a ctivities and WC were not statistically significant between the male and female subgroups (*P* > 0.05). Because of these significant differences between males and females (*P* < 0.05), we performed the main analyses separately by sex.

**Table1:** Characteristics of participants with full samples(*N* = 4423)

Variables	Male	Female	Total	*t/χ*^*2*^	*P*
	**N (%)**	**N (%)**	**N (%)**		
N	2038(46.10)	2385(53.90)	4423(100)		
Age(years)					
45–54	658(32.29)	1099(46.08)	1757(39.72)	112.022	0.000
55–64	835(40.97)	896(37.57)	1731(39.14)		
65–74	431(21.15)	310(13.00)	741(16.75)		
≥ 75	114(5.59)	80(3.35)	194(4.39)		
Education					
Illiterate	263(12.90)	943(39.54)	1206(27.27)	404.201	0.000
Less than elementary school	1509(74.04)	1278(53.58)	2787(63.01)		
High school	190(9.32)	128(5.37)	318(7.19)		
Above vocational school	76(3.73)	36(1.51)	112(2.53)		
Marital status					
Single	153(7.51)	257(10.78)	410(9.27)	13.957	0.000
Married	1885(92.49)	2128(89.22)	4013(90.73)		
Current residence					
Rural	1924(94.41)	2231(93.54)	4155(93.94)	1.439	0.230
Urban	114(5.59)	154(6.46)	268(6.06)		
Current smoking					
No	505(24.78)	2232(93.58)	2737(61.88)	2205.999	0.000
Former smoke	294(14.43)	35(1.47)	329(7.44)		
Current smoke	1239(60.79)	118(4.95)	1357(30.68)		
Alcohol drinking					
No	877(43.03)	2081(87.25)	2958(66.88)	1009.224	0.000
Less than once a month	228(11.19)	124(5.20)	352(7.96)		
More than once a month	933(45.78)	180(7.55)	1113(25.16)		
Taking activities					
No	1000(49.07)	1238(51.91)	2238(50.60)	3.546	0.060
Yes	1038(50.93)	1147(48.09)	2185(49.40)		
Having regular exercises					
No exercise	1266(62.12)	1409(59.08)	2675(60.48)	4.599	0.100
Less than exercises	398(19.53)	517(21.68)	915(20.69)		
Regular exercises	374(18.35)	459(19.25)	833(18.83)		
Chronic diseases(counts)					
0	841(41.27)	921(38.62)	1762(39.84)	3.230	0.199
1–2	983(48.23)	1200(50.31)	2183(49.36)		
3–14	214(10.50)	264(11.07)	478(10.81)		
WC	82.94 ± 8.85	83.46 ± 9.46	83.22 ± 9.46	-1.861	0.063
BMI	22.32 ± 3.20	23.37 ± 3.82	22.89 ± 3.82	-10.029	0.000
WHtR	0.51 ± 0.05	0.55 ± 0.06	0.53 ± 0.06	-22.640	0.000
VAI	3.56 ± 3.88	5.35 ± 4.89	4.52 ± 4.89	-13.580	0.000
ABSI	8.20 ± 0.51	8.29 ± 0.59	8.25 ± 0.59	-5.116	0.000
BRI	3.54 ± 1.01	4.32 ± 1.28	3.96 ± 1.28	-22.647	0.000
LAP	25.67 ± 26.90	36.99 ± 29.77	31.77 ± 29.77	-13.287	0.000
CI	1.26 ± 0.08	1.28 ± 0.09	1.27 ± 0.09	-8.703	0.000
CVAI	85.47 ± 43.06	94.52 ± 40.32	90.35 ± 40.32	-7.175	0.000
TyG index	8.54 ± 0.62	8.62 ± 0.60	8.58 ± 0.60	-4.149	0.000
TyG-BMI	191.14 ± 34.20	201.89 ± 38.57	196.93 ± 38.57	-9.824	0.000
TyG-WC	710.13 ± 105.26	720.61 ± 106.57	715.78 ± 106.57	-3.278	0.001
TyG -WHtR	4.34 ± 0.62	4.71 ± 0.69	4.54 ± 0.69	-18.778	0.000

*WC* Waist circumference, *BMI* Body mass index, *WHtR* Waist to height ratio, *VAI* Visceral adiposity index, *ABSI* A body shape index, *BRI* Body roundness index, *LAP* Lipid accumulation product, *CVAI* Chinese visceral adiposity index, *CI* Conicity index, *TyG* Triglyceride and glucose index, *TyG-BMI* TyG related to BMI, *TyG-WC* TyG related to WC, *TyG-WHtR* TyG related to WHtR

Table 2 shows the baseline characteristics of the study participants with and without future HTN by sex. According to the study's findings, during the four years, the incident rate of males with hypertension was 22.08%, while the incident rate of women with hypertension was 17.82%. Men with HTN had significant differences in age, current residence, smoking, alcohol consumption, WC, BMI, WHtR, VAI, ABSI, BRI, LAP, CI, CVAI, TyG index, TyG-BMI, TyG-WC and TyG-WHtR (*P* < 0.05); women with HTN had significant differences in age, education, marital status, WC, BMI, WHtR, VAI, ABSI, BRI, LAP, CI, CVAI, TyG index, TyG-BMI, TyG-WC and TyG-WHtR (*P* < 0.05). Data were analyzed using chi-square tests, and then Bonferroni adjustments were used to reassess the significance levels in Table 2. Among men, the incidence of hypertension was higher in the 65–74 years-old group compared with the 45–54 years-old group, the 55–64 years-old group, and the ≥ 75 years-old group (Using Bonferroni correction, run 6 comparisons, critical value *P* = 0.008, post-adjustment *P* < 0.008). The incidence was higher in participants who were former smokers compared to those who were current smokers (Using Bonferroni correction, run 3 comparisons, critical value *P* = 0.017, post-adjustment *P* < 0.017). Participants who drank alcohol more than once a month had a higher incidence compared to those who drank less than once a month (Using Bonferroni correction, run 3 comparisons, critical value *P* = 0.017, post-adjustment *P* < 0.017). The incidence rate of male living in urban is higher (*P* < 0.05). Among women, the incidence of hypertension was higher in the ≥ 75 years-old group than in the 45–54 years-old group, 55–64 years-old group, and 65–74 years-old group (Using Bonferroni correction, run 6 comparisons, critical value *P* = 0.008, post-adjustment *P* < 0.008). Illiterate participants were more likely to develop hypertension than participants with less than elementary or high school levels of literacy (Using Bonferroni correction, run 6 comparisons, critical value *P* = 0.008, post-adjustment *P* < 0.008). Married women have a higher incidence than single women (*P* < 0.05).

**Table2:** Baseline characteristics of the study participants with and without future HTN by sex

Variables	Male (*N* = 2038)		*χ2*	*P*	Female (*N* = 2385)		*χ2*	*P*
**N (%)**	**With HTN N (%)**	**Without HTN N (%)**			**With HTN N (%)**	**Without HTN N (%)**		
N	450(22.08)	1588(77.92)			425(17.82)	1960(82.18)		
Age(years)								
45–54	119(26.44)	539(33.94)	27.381	0.000	148(34.82)	951(48.52)	58.993	0.000
55–64	168(37.33)	667(42.00)			160(37.65)	736(37.55)		
65–74	129(28.67)	302(19.02)			86(20.24)	224(11.43)		
≥ 75	34(7.56)	80(5.04)			31(7.29)	49(2.50)		
Education								
Illiterate	60(13.33)	203(12.78)	3.926	0.270	201(47.29)	742(37.86)	16.404	0.001
Less than elementary school	336(74.67)	1173(73.87)			207(48.71)	1071(54.64)		
High school	33(7.33)	157(9.89)			13(3.06)	115(5.87)		
Above vocational school	21(4.67)	55(3.46)			4(0.94)	32(1.63)		
Marital status								
Single	39(8.67)	114(7.18)	1.118	0.290	73(17.18)	184(9.39)	22.037	0.000
Married	411(91.33)	1474(92.82)			352(82.82)	1776(90.61)		
Current residence								
Rural	413(91.78)	1511(95.15)	7.556	0.006	402(94.59)	1829(93.32)	0.935	0.333
Urban	37(8.22)	77(4.85)			23(5.41)	131(6.68)		
Current smoking								
No	117(26.00)	388(24.43)	6.831	0.033	397(93.41)	1835(93.62)	1.489	0.475
Former smoke	80(17.78)	214(13.48)			4(0.94)	31(1.58)		
Current smoke	253(56.22)	986(62.09)			24(5.65)	94(4.80)		
Alcohol drinking								
No	187(41.56)	690(43.45)	9.306	0.010	371(87.29)	1710(87.24)	0.853	0.653
Less than once a month	35(7.78)	193(12.15)			19(4.47)	105(5.36)		
More than once a month	228(50.67)	705(44.40)			35(8.24)	145(7.40)		
Taking activities								
No	220(48.89)	780(49.12)	0.007	0.931	221(52.00)	1017(51.89)	0.002	0.967
Yes	230(51.11)	808(50.88)			204(48.00)	943(48.11)		
Having regular exercises								
No exercise	269(59.78)	997(62.78)	2.008	0.366	254(59.76)	1155(58.93)	2.092	0.351
Less than exercises	98(21.78)	300(18.89)			99(23.29)	418(21.33)		
Regular exercises	83(18.44)	291(18.32)			72(16.94)	387(19.74)		
Chronic diseases(counts)								
0	183(40.67)	658(41.44)	0.687	0.709	156(36.71)	765(39.03)	0.992	0.609
1–2	215(47.78)	768(48.36)			218(51.29)	982(50.10)		
3–14	52(11.56)	162(10.20)			51(12.00)	213(10.87)		
WC	85.02 ± 9.39	82.35 ± 8.61	-5.413	0.000	86.12 ± 9.65	82.88 ± 9.32	-6.447	0.000
BMI	22.88 ± 3.38	22.16 ± 3.13	-4.047	0.000	24.16 ± 4.05	23.20 ± 3.74	-4.686	0.000
WHtR	0.52 ± 0.05	0.50 ± 0.05	-6.309	0.000	0.56 ± 0.06	0.54 ± 0.06	-7.225	0.000
VAI	4.08 ± 4.49	3.41 ± 3.68	-2.914	0.004	6.06 ± 5.47	5.19 ± 4.75	-3.033	0.003
ABSI	8.28 ± 0.49	8.18 ± 0.51	-3.557	0.000	8.39 ± 0.62	8.27 ± 0.58	-3.803	0.000
BRI	3.80 ± 1.07	3.47 ± 0.98	-6.020	0.000	4.73 ± 1.35	4.23 ± 1.25	-7.260	0.000
LAP	31.79 ± 32.33	23.93 ± 24.88	-4.768	0.000	44.46 ± 34.12	35.37 ± 28.50	-5.119	0.000
CI	1.28 ± 0.08	1.26 ± 0.08	-5.109	0.000	1.31 ± 0.09	1.28 ± 0.09	-5.620	0.000
CVAI	96.58 ± 46.27	82.33 ± 41.58	-5.894	0.000	109.89 ± 40.74	91.19 ± 39.46	-8.802	0.000
TyG index	8.67 ± 0.68	8.51 ± 0.60	-4.664	0.000	8.74 ± 0.64	8.59 ± 0.59	-4.278	0.000
TyG-BMI	198.92 ± 36.74	188.93 ± 33.13	-5.201	0.000	211.58 ± 41.67	199.78 ± 37.54	-5.383	0.000
TyG-WC	739.09 ± 114.73	701.93 ± 100.95	-6.221	0.000	753.88 ± 111.72	713.40 ± 104.05	-7.174	0.000
TyG -WHtR	4.52 ± 0.67	4.29 ± 0.59	-6.742	0.000	4.94 ± 0.72	4.66 ± 0.67	-7.785	0.000

*WC* Waist circumference, *BMI* Body mass index, *WHtR* Waist to height ratio, *VAI* Visceral adiposity index, *ABSI* A body shape index, *BRI* Body roundness index, *LAP* Lipid accumulation product, *CVAI* Chinese visceral adiposity index, *CI* Conicity index, *TyG* Triglyceride and glucose index, *TyG-BMI* TyG related to BMI, *TyG-WC* TyG related to WC, *TyG-WHtR* TyG related to WHtR

**Fig. 1:**
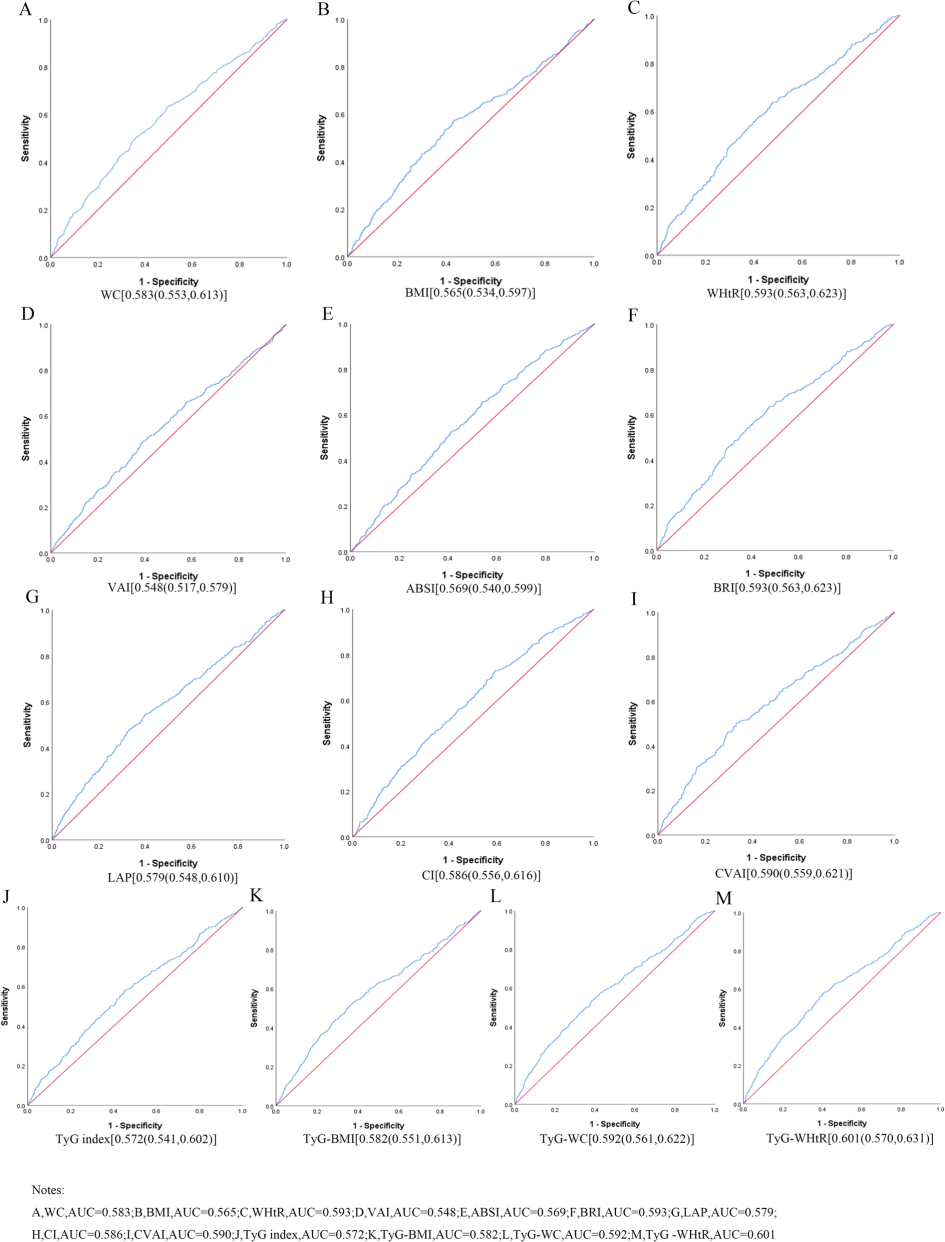
The ROC curves of each indicator in the prediction of HTN risk in males

**Fig. 2:**
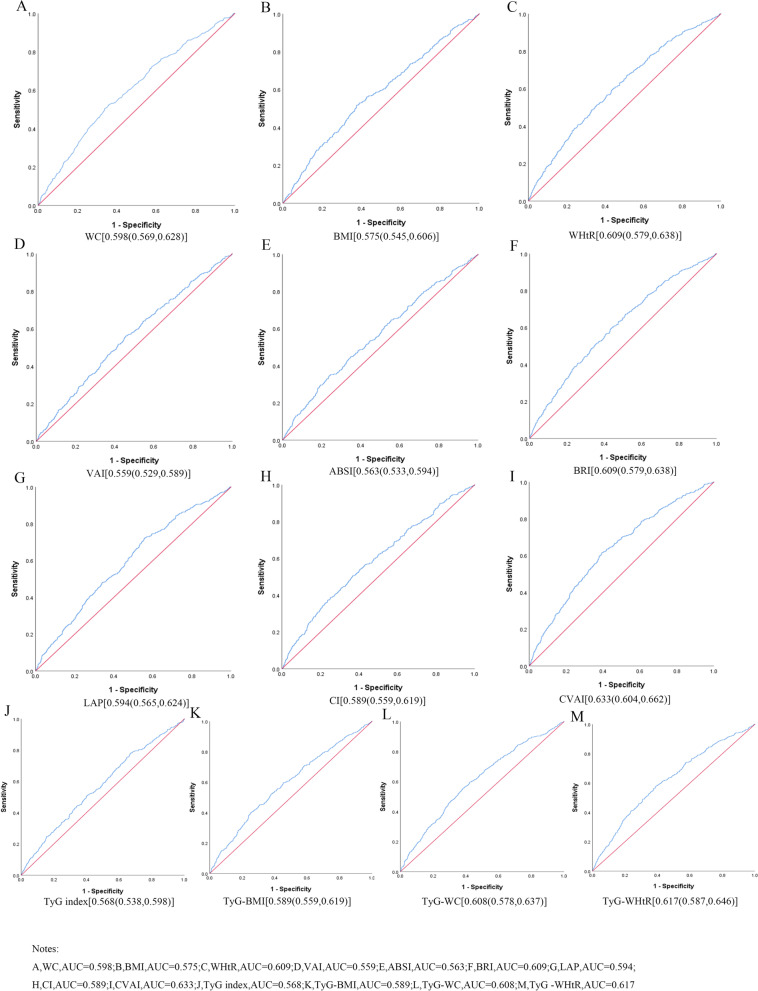
The ROC curves of each indicator in the prediction of HTN risk in females

Table 3 shows the cut-off value between the area under curve, sensitivity, and specificity for obesity and lipid-related indices to detect HTN by sex. The ROC curves of each indicator in the prediction of HTN risk in men and women are shown in Figs. 1 and 2 respectively. The significance of the area under the curve is that an area greater than 0.9 indicates high accuracy, 0.7–0.9 indicates moderate accuracy, 0.5–0.7 indicates low accuracy, and 0.5 indicates a chance result [47]. As shown in the table and figures, among men, the WHtR was the best predictor of HTN in the middle-aged and elderly male population (AUC = 0.593, SE = 0.015, 95% CI [0.563,0.623], and optimal cutoffs = 0.501). Meanwhile, BRI (AUC = 0.593, SE = 0.015, 95%CI [0.563,0.623], and optimal cutoffs = 3.371) had similar predictive values. Moreover, among women, CVAI was the most accurate predictor of HTN in middle-aged and elderly women (AUC = 0.633, SE = 0.015, 95%CI [0.604,0.662], and optimal cutoffs = 99.405). All of the above indicators were statistically different (*P* < 0.05). From the overall data, the AUC values of the above thirteen indicators were higher than 0.5, indicating that they have predictive value for hypertension.

**Table 3:** Cut-off between area under curve, sensitivity and specificity for obesity- and lipid-related indices to detect HTN by sex

*N* = 4423	WC	BMI	WHtR	VAI	ABSI	BRI	LAP	CI	CVAI	TyG index	TyG-BMI	TyG-WC	TyG -WHtR
**Male**													
Area under curve	0.583	0.565	0.593	0.548	0.569	0.593	0.579	0.586	0.590	0.572	0.582	0.592	0.601
Std. Error	0.016	0.016	0.015	0.016	0.015	0.015	0.016	0.015	0.016	0.016	0.016	0.016	0.015
95%CI	0.552,0.613	0.534,0.597	0.563,0.623	0.517,0.579	0.540,0.599	0.563,0.623	0.548,0.61	0.556,0.616	0.559,0.621	0.541,0.602	0.551,0.613	0.561,0.622	0.570,0.631
*P*-value	0.000	0.000	0.000	0.002	0.000	0.000	0.000	0.000	0.000	0.000	0.000	0.000	0.000
Optimal cutoffs	84.250	22.221	0.501	2.849	8.120	3.371	23.533	1.237	94.912	8.485	193.330	716.537	4.335
J-Youden	0.143	0.142	0.162	0.099	0.117	0.162	0.149	0.140	0.173	0.129	0.156	0.152	0.175
Sensitivity (%)	50.20%	57.60%	63.60%	48.70%	65.80%	63.60%	47.60%	**73.10%**	50.40%	58.40%	53.30%	53.60%	58.20%
Specificity (%)	64.10%	56.60%	52.60%	61.20%	45.90%	52.60%	67.30%	40.90%	66.90%	54.50%	62.30%	61.60%	59.30%
( +) Likelihood ratio	1.398	1.327	1.342	1.255	1.216	1.342	1.456	1.237	1.523	1.284	1.414	1.396	1.43
(-) Likelihood ratio	0.777	0.749	0.692	0.838	0.745	0.692	0.779	0.658	0.741	0.763	0.750	0.753	0.705
**Female**													
Area under curve	0.598	0.575	0.609	0.559	0.563	0.609	0.594	0.589	0.633	0.568	0.589	0.608	0.617
Std. Error	0.015	0.015	0.015	0.015	0.016	0.015	0.015	0.015	0.015	0.015	0.015	0.015	0.015
95%CI	0.569,0.628	0.545,0.606	0.579,0.638	0.529,0.589	0.533,0.594	0.579,0.638	0.565,0.624	0.559,0.619	0.604,0.662	0.538,0.598	0.559,0.619	0.578,0.637	0.587,0.646
*P*-value	0.000	0.000	0.000	0.000	0.000	0.000	0.000	0.000	0.000	0.000	0.000	0.000	0.000
Optimal cutoffs	86.650	23.962	0.548	3.970	8.611	4.295	24.697	1.307	99.405	8.293	219.635	736.702	4.781
J-Youden	0.166	0.142	0.160	0.105	0.108	0.160	0.165	0.138	0.222	0.110	0.147	0.175	0.189
Sensitivity (%)	50.40%	51.80%	59.50%	56.20%	34.60%	59.50%	**72.50%**	50.60%	61.60%	**78.10%**	40.20%	55.80%	58.10%
Specificity (%)	66.20%	62.40%	56.50%	54.30%	76.20%	56.50%	44.00%	63.20%	60.60%	32.90%	74.50%	61.70%	60.80%
( +) Likelihood ratio	1.491	1.378	1.368	1.230	1.454	1.368	1.295	1.375	1.563	1.164	1.576	1.457	1.482
(-) Likelihood ratio	0.749	0.772	0.717	0.807	0.858	0.717	0.625	0.782	0.634	0.666	0.803	0.716	0.689

*WC* Waist circumference, *BMI* Body mass index, *WHtR* Waist to height ratio, *VAI* Visceral adiposity index, *ABSI* A body shape index, *BRI* Body roundness index, *LAP* Lipid accumulation product, *CVAI* Chinese visceral adiposity index, *CI* Conicity index, *TyG* Triglyceride and glucose index, *TyG-BMI* TyG related to BMI, *TyG-WC* TyG related to WC, *TyG-WHtR* TyG related to WHtR

**Fig. 3:**
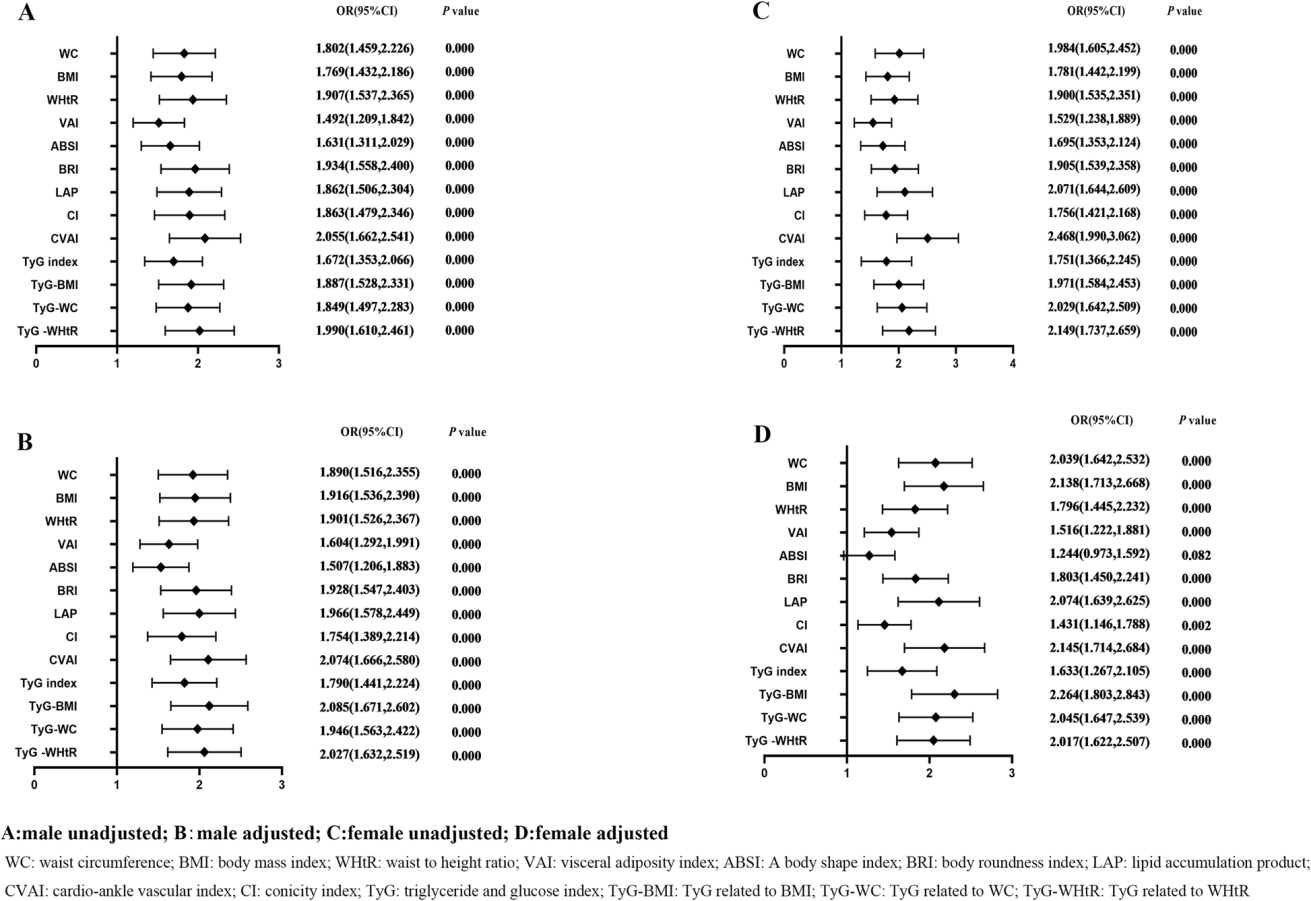
Forest diagram of or value before and after adjustment of confounding factors for males and females

Table 4 shows the associations of obesity- and lipid-related indices with HTN. According to the values in Table 3, 13 obesity- and lipid-related indices were transformed into two-category variables in this investigation. Table 4 is based on the transformed variables. A larger OR, in general, suggests a higher risk factor. Both before and after adjusting for age, education, marital status, current residence, current smoking, alcohol drinking, social activity, exercise, and chronic disease counts, the odds of elevated BP increased progressively with increasing obesity and units of lipid measurement for both men and women. Each unit rise in WHtR, for example, was related with a 1.036-fold (95% CI:1.024,1.049) increase in the likelihood of developing hypertension in males. Each unit increase in CVAI was linked to a 1.009-fold increase in the likelihood of developing hypertension in women (95% CI:1.007,1.012). In addition, except for female ABSI, which had no significant significance after adjustment of confounding factors (*P* > 0.05), all other indexes had statistical significance after adjustment of confounding factors (*P* < 0.05). Figure 3 shows the forest diagram of or value before and after adjustment of confounding factors for males and females.

**Table 4:** Associations of obesity- and lipid-related indices with HTN and its components

HTN	WC	BMI	WHtR	VAI	ABSI	BRI	LAP	CI	CVAI	TyG index	TyG-BMI	TyG-WC	TyG -WHtR
**Male**													
Unadjusted OR (95% CI)	1.802(1.459,2.226)	1.769(1.432,2.186)	1.907(1.537,2.365)	1.492(1.209,1.842)	1.631(1.311,2.029)	1.934(1.558,2.400)	1.862(1.506,2.304)	1.863(1.479,2.346)	2.055(1.662,2.541)	1.672(1.353,2.066)	1.887(1.528,2.331)	1.849(1.497,2.283)	1.990(1.610,2.461)
*P* value	0.000	0.000	0.000	0.000	0.000	0.000	0.000	0.000	0.000	0.000	0.000	0.000	0.000
Adjusted OR (95% CI)	1.890(1.516,2.355)	1.916(1.536,2.390)	1.901(1.526,2.367)	1.604(1.292,1.991)	1.507(1.206,1.883)	1.928(1.547,2.403)	1.966(1.578,2.449)	1.754(1.389,2.214)	2.074(1.666,2.580)	1.790(1.441,2.224)	2.085(1.671,2.602)	1.946(1.563,2.422)	2.027(1.632,2.519)
*P* value	0.000	0.000	0.000	0.000	0.000	0.000	0.000	0.000	0.000	0.000	0.000	0.000	0.000
**Female**													
Unadjusted OR (95% CI)	1.984(1.605,2.452)	1.781(1.442,2.199)	1.900(1.535,2.351)	1.529(1.238,1.889)	1.695(1.353,2.124)	1.905(1.539,2.358)	2.071(1.644,2.609)	1.756(1.421,2.168)	2.468(1.990,3.062)	1.751(1.366,2.245)	1.971(1.584,2.453)	2.029(1.642,2.509)	2.149(1.737,2.659)
*P* value	0.000	0.000	0.000	0.000	0.000	0.000	0.000	0.000	0.000	0.000	0.000	0.000	0.000
Adjusted OR (95% CI)	2.039(1.642,2.532)	2.138(1.713,2.668)	1.796(1.445,2.232)	1.516(1.222,1.881)	1.244(0.973,1.592)	1.803(1.450,2.241)	2.074(1.639,2.625)	1.431(1.146,1.788)	2.145(1.714,2.684)	1.633(1.267,2.105)	2.264(1.803,2.843)	2.045(1.647,2.539)	2.017(1.622,2.507)
*P* value	0.000	0.000	0.000	0.000	0.082	0.000	0.000	0.002	0.000	0.000	0.000	0.000	0.000

*WC* Waist circumference, *BMI* Body mass index, *WHtR* Waist to height ratio, *VAI* Visceral adiposity index, *ABSI* A body shape index, *BRI* Body roundness index, *LAP* Lipid accumulation product, *CVAI* Chinese visceral adiposity index, *CI* Conicity index, *TyG* Triglyceride and glucose index, *TyG-BMI* TyG related to BMI, *TyG-WC* TyG related to WC, *TyG-WHtR* TyG related to WHtR, Odds ratios were adjusted for age, educational levels, marital status, live place, current smoking, alcohol drinking, activities, exercises, chronic diseases

## Discussion

Due to its great incidence and challenging management, hypertension has turned into a threat to public health. Around 1.4 billion individuals worldwide have hypertension, which has increased to 31.3% of the population between 2000 and 2010 [6]. Because of disparities in knowledge, treatment, and capacity to control hypertension, the incidence of hypertension has dropped by 2.6% in high-income nations while increasing by 7.7% in low- and middle-income countries. In a study in China [7], the incident rate of hypertension among Chinese adults was 23.2% from 2012 to 2015, even though the Chinese government has taken many proactive measures, such as providing Essential Public Health Services to screen for chronic diseases and increase the coverage of health insurance [49], limiting salt intake [50], the effect is still not satisfactory [4].

In obese individuals, adipocyte dysfunction contributes to vascular and systemic insulin resistance, as well as malfunction of the sympathetic nervous system and the renin–angiotensin–aldosterone system. It causes contraction of vascular smooth muscle, retention of water and sodium, and pressure increase. The long-term increase of cardiac output and blood volume will lead to the increase of systemic vascular resistance. The development of obesity-associated hypertension is also influenced by structural and functional changes in the kidney, such as the activation of intrarenal angiotensin II [51–53]. It has been shown that obese compared with non-obese hypertensive individuals exhibit higher renal sodium reabsorption, and perhaps in association with this functional change, higher total body water, plasma volume, and intracellular body water [54]. Visceral adipose tissue is also resistant to insulin and leptin and is the site of altered secretion of molecules and hormones such as adiponectin, leptin, resistin, tumor necrosis factor and interleukin-6, which exacerbate cardiovascular disease associated with obesity [55].

The incidence rate of hypertension can currently be predicted by the obesity index, according to numerous research. However, the findings of academic studies remain contradictory when taking into account racial factors [56] and variations in body composition. BMI is used as the most widely used anthropometric method in clinical and epidemiological studies [57, 58] to define obesity and overweight. However, BMI can't show the distribution characteristics of fat, and it is influenced by age, sex, and race [59], which also makes more scholars question its accuracy, and put forward many new anthropometric indicators.

In this study, the prediction ability of WHtR and Tyg-WHtR is modest. WHtR is a better indicator for detecting abdominal obesity than BMI and WC because it takes WC and height into account as a whole [60]. A study from Thailand [61] compared nine obesity indices with hypertension and showed that WHtR was the most practical measure of obesity associated with hypertension in both men and women.The WHtR recommended value for predicting hypertension is set to 0.5 [62]. Our research results show that WHtR = 0.501 has the strongest prediction effect, which is closer to the recommended value. It is basically consistent with the research results of other scholars [63, 64]. But some scholars pointed out that the people selected by this standard are mostly Asian people, and perhaps in non-Asian areas, this standard is not applicable due to the differences of race and human body composition [63]. A notion initially introduced by Ko et al. [65]is known as the Tyg index-related parameters, which integrate the TyG index with WC, BMI, and WHtR. Later, it was noted that [66] the combined index of TyG index and obesity index was superior to the single index and more useful in predicting the risk of hypertension development.

In our large national cohort study, we used ROC to determine the cut-off values for obesity and lipid-related indicators. The results of the study showed that women had higher cut-off values for the indicators compared to men. This may be related to the different body composition of men and women [67]. According to the values in Table 3, 13 obesity- and lipid-related indices were transformed into two-category variables in this investigation. The modified variables are used in Table 4. A larger OR, in general, suggests a higher risk factor. Before adjusting for potential confounders, the results of multivariate logistic regression models showed that the ORs of obesity and lipid-related indices were mostly higher in women than in men. However, after adjusting for potential confounders, the ORs of females decreased more than those of males, and even the ORs of some indicators were smaller than those of males. This can be partially explained by the fact that women may be more susceptible to potential confounders. The effect of these potential confounders on hypertension needs to be further investigated.

There are a few limitations to be aware of. First, the relationship should be studied prospectively. Second, we only considered the identified confounders. However, some unknown factors still existed. Thirdly, in order to avoid white-coat hypertension and occult hypertension, it is suggested to use out-of-office blood pressure assessment methods, such as ambulatory BP monitoring (ABPM) or home BP monitoring (HBPM), but our research method does not take this situation into account. Fourth, our results showed that the AUC values mostly hovered around 0.6, with low diagnostic accuracy. In future studies, we need to try to combine two or more indices to see if the diagnostic accuracy can be improved. Fifth, in our study, the sensitivity and specificity of these WHtR and Tyg-WHtR were indeed not high, so they were only modest predictors of hypertension, but in terms of area under the curve, they were indeed more cost-effective than the others. The advantages of this study are as follows: First, cohort research design, relatively large sample size and gender-specific analysis method provide guarantee for determining the causal relationship. Second, the study's huge sample of 4423 middle-aged and older Chinese is another important strength. The analytical approach that managed the various confounders is the last advantage.

## Conclusion

In this cohort study, WHtR and Tyg-WHtR are the most cost-effective indicators with moderate predictive value of the development of HTN. In addition, when WHtR = 0.501 or TYg-WHtR = 4.335, the effect of predicting the incidence risk of men is the best, and when WHtR = 0.548 or TYg-WHtR = 4.781, the effect of predicting the incidence risk of women is the best.

## Data Availability

The datasets generated and/or analyzed during the current study are publicly available in the http://charls.pku.edu.cn/index.html repository.

## Abbreviations

CHARLS: China Health and Retirement Longitudinal Study
HTN: Hypertension
WC: Waist circumference
BMI: Body mass index
WHtR: Waist-height ratio
VAI: Visceral adiposity index
ABSI: A body shape index
BRI: Body roundness index
LAP: Lipid accumulation product index
CI: Conicity index
CVAI: Chinese visceral adiposity index
TyG-index: Triglyceride-glucose index
ROC: Receiver operating characteristic curve
AUC: Area under curve
HDL-C: High-density lipoprotein cholesterol
BP: Blood pressure
SBP: Systolic blood pressure
DBP: Diastolic blood pressure
TG: Triglycerides
FPG: Fasting plasma glucose
SPSS: Statistical Product Service Solutions
ORs: Odds ratios
CI: Confidence interval
SD: Standard deviation
